# Elevated cyclin A associated kinase activity promotes sensitivity of metastatic human cancer cells to DNA antimetabolite drug

**DOI:** 10.3892/ijo.2015.3037

**Published:** 2015-06-08

**Authors:** JIN WANG, HAILIN YIN, ASHWINI PANANDIKAR, VARSHA GANDHI, SUBRATA SEN

**Affiliations:** 1 Department of Translational Molecular Pathology, The University of Texas M.D. Anderson Cancer Center, Houston, TX 77030, USA; 2 Department of Experimental Therapeutics, The University of Texas M.D. Anderson Cancer Center, Houston, TX 77030, USA; 3 Program in Human and Molecular Genetics, University of Texas Graduate School of Biomedical Sciences, Houston, TX 77030, USA

**Keywords:** drug resistance, cyclin A, N(phosphonoacetyl)-L-aspartate, apoptosis pathway

## Abstract

Drug resistance is a major obstacle in successful systemic therapy of metastatic cancer. We analyzed the involvement of cell cycle regulatory proteins in eliciting response to N (phosphonoacetyl)-L-aspartate (PALA), an inhibitor of *de novo* pyrimidine synthesis, in two metastatic variants of human cancer cell line MDA-MB-435 isolated from lung (L-2) and brain (Br-1) in nude mouse, respectively. L-2 and Br-l cells markedly differed in their sensitivity to PALA. While both cell types displayed an initial S phase delay/arrest, Br-l cells proliferated but most L-2 cells underwent apoptosis. There was distinct elevation in cyclin A, and phosphorylated Rb proteins concomitant with decreased expression of bcl-2 protein in the PALA treated L-2 cells undergoing apoptosis. Markedly elevated cyclin A associated and cdk2 kinase activities together with increased E2F1-DNA binding were detected in these L-2 cells. Induced ectopic cyclin A expression sensitized Br-l cells to PALA by activating an apoptotic pathway. Our findings demonstrate that elevated expression of cyclin A and associated kinase can activate an apoptotic pathway in cells exposed to DNA antimetabolites. Abrogation of this pathway can lead to resistance against these drugs in metastatic variants of human carcinoma cells.

## Introduction

Drug resistance is a major obstacle in successful chemotherapy of metastatic breast cancer and most patients with drug resistant metastatic cancer succumb to the disease. Therefore, it is important to investigate the genetic basis of drug resistance in metastatic cancer cells to design effective therapy of advanced stage disease and improve their clinical outcome. Increased genetic instability leads to progressive metastasis of tumors with simultaneous acquisition of resistance to chemotherapeutic drugs. Recent advances in understanding the mode of action of anticancer drugs indicate that regardless of their diverse nature, most of them elicit apoptosis in the target cells (1,2). The genetic changes which inactivate apoptotic pathways during tumor development make tumor cells resistant to drugs. Apoptosis is associated with activation of a number of genes that mediate the transition from quiescence to proliferative growth (3,4). Premature activation of a subset of protein kinases, normally active in mitosis, has been implicated as critical events in the apoptosis process (5,6).

Cyclin proteins associating with and activating cyclin dependent kinases are central to the control of cell proliferation in eukaryotic cells. These proteins are involved at critical nodal points, shared by pathways regulating both cellular proliferation as well as apoptosis (7–13). Phosphorylation of pRb by cyclin D/cdk4, cyclin E/cdk2 and/or cyclin A/cdk2 during Gl/S transition releases the transcription factor E2F1 which directs the timely expression of cell cycle controlling genes regulating initiation and orderly progression of S phase (14,15). Cyclin A kinase through binding and phosphorylation of the transcription factor E2F1 suppresses expression of specific S phase genes. The regulation of E2F1 DNA binding and transactivation has been proposed to represent an S phase checkpoint which when disrupted due to decreased cyclin A/cdk2 activity results in S phase delay/arrest followed by regrowth or apoptosis (16). Enhanced DNA binding of hypophosphorylated E2F1 has been correlated with increased sensitivity of human tumor cells to cytotoxic drug and ionizing radiation treatments (17,18).

Two metastatic variants of the human cancer cell line (MDA-MB-435) isolated from lung and brain metastasis in nude mice (19) demonstrate markedly different levels of sensitivity to antimetabolite chemotherapeutic drugs. In this study, we investigated the involvement of cell cycle regulatory proteins in eliciting differential response in these two variants to the antimetabolite drug PALA. PALA is a potent inhibitor of aspartate transcarbamoyl transferase, the enzyme catalyzing the second step of *de novo* pyrimidine biosynthesis.

We observed elevation of cyclin A expression and activation of its catalytic subunit kinase in the drug sensitive L-2 cells undergoing apoptosis but not in the resistant Brl cells. Further, we demonstrated that induced ectopic expression of cyclin A was sufficient to cause apoptosis in the resistant Br1 cells when exposed to PALA. In cells undergoing apoptosis, elevated cyclin A expression and kinase activities also correlated with increased E2Fl DNA binding activity. Therefore, this study provides evidence that apoptotic response in antimetabolite drug-treated tumor cells involves enhanced cyclin A/cdk2 activity concomitant with increased E2Fl DNA binding activity. Taken together these results suggest that cyclin A and associated kinase activity are regulators of a checkpoint response that is activated in drug-treated cells leading to induction of apoptosis.

## Materials and methods

### Cell lines and drug selection

Br-l and L-2 cell lines established from metastasis in nude mouse injected with the human tumor cell line MDA-MB-435 were provided by Dr Janet E. Price of the Department of Cancer Biology, The University of Texas M.D. Anderson Cancer Center. MDA-MB-435, isolated from plural effusion of a 31-year-old breast cancer patient, was later reported to show similarity with melanocyte/melanoma cells based on gene expression profiling data. The Br-l cell line was established from a brain metastasis while L-2 cells were selected by two cycles of growth and metastasis to lung in nude mice (19). Cell lines were maintained in Dulbecco’s modified Eagle’s medium supplemented with 10% dialyzed FBS (Gibco, Grand Island, NY, USA). The cells were grown on plastic and incubated in 5% CO_2_ in air at 37°C in a humidified incubator. Three independent clones isolated from the L-2 (L-2, L-2-1, L-2-2) and Br-1 (Brl-3prl, Brl-3pr2, Brl3pr3) were utilized in this study. Population doubling time, for each of these cell lines were estimated to be ~24 h. Drug resistance levels and proliferative response to drug treatment among the clonal isolates from each cell type variant were very similar.

Cell lines were tested for their potential to acquire resistance against the DNA antimetabolite drug PALA. Frequency of drug resistant cells developing at 5xLD_50_ concentration of the drug were <10^−5^ for L-2 and <10^−3^ for Br-1 cells. PALA was obtained from Drug synthesis Branch (Division of Cancer Treatment, National Cancer Institute). At 20xLD_50_, Br-1 cells gave rise to resistant colonies but L-2 cells did not. Further experiments to study early proliferative response and cell cycle regulatory protein expression were done with cells exposed to 20xLD_50_ of PALA (300 μM).

### Drug treatment

L-2 and Brl-3prl cells were plated at a density of 1-2×10^6^ and 48 h later 300 μM of PALA was added. Cells were initially harvested after 12, 24 and 48 h of PALA treatment for flow cytometry analysis and oligonucleosomal DNA analyses. In another set of experiments, cells treated for 48 h were washed, re-plated in drug-free medium and harvested at 0, 4, 10, 24 and 48 h for flow cytometry, oligonucleosomal DNA analysis and protein analysis. Cells harvested immediately after 48 h of PALA at 0 h were considered as those representing the control time point.

### Growth rate analysis

Exponentially growing L-2 and Brl-3prl were plated in 60-mm dishes at a cell density of 3×10^5^. After 48 h, regular medium was replaced with medium containing 300 μM of PALA (20xLD_50_). PALA was washed off after 48 h and regular medium was added. Cells were counted from day 0 through day 5 for every 24 h with trypan blue staining.

### Flow cytometry analysis

Approximately 1×10^6^ cells were washed with phosphate buffered saline (PBS), fixed overnight in 70% ethanol, stained with propidium iodide (final concentration was 0.01μg/ml in PBS and analyzed on a FACScan cytometer (Becton Dickinson). Resolution of G1, S and G2/M phases was done with LYSIS II analysis software (Becton Dickinson).

### Effect of PALA on cellular nucleotides

To determine the PALA-mediated perturbation in the ribonucleotide pools, each cell line was incubated with 300 μM PALA for 48 h. After treatment cells were washed with phosphate-buffered saline, enumerated with Coulter counter analyzer, and mean cell volume was determined with the Coulter channelyzer (Coulter Electronics Inc., Hialeah, FL, USA). Nucleotides were extracted from cells by standard procedures using HC104 before (control) and 48 h after incubation with 300 μM PALA. Ribonucleotides were separated using an anion-exchange Partisil-10 SAX (Waters Corp., Milford, MA, USA) column by high-pressure liquid chromatography (45). The intracellular concentration was calculated and expressed as the quantity of nucleotides contained in the extract from a given number of cells of a determined mean volume. This calculation assumes that nucleotides are uniformly distributed in total cell water. In general, the lower limit of sensitivity of this assay was 50 pmol in an extract of 1×10^7^ cells, corresponding to a cellular concentration of ~2.5 μM.

### Oligonucleosomal DNA analysis

For DNA fragmentation assay, both adherent and non-adherent cells were washed in PBS and lysed in 100 μl ice cold PBS and 400 μl lysis buffer (Tris-EDTA, 0.1% Triton X-100) on ice for 20 min. The supernatant containing fragmented DNA was isolated by centrifuging and precipitating with NaCl and isopropanol at −20°C overnight. The pellets were resuspended in 150 μl of buffer containing 10 mM Tris pH 7.8, 25 mM EDTA, 0.5% SDS and 100 μg of proteinase K at 37°C overnight. The samples were extracted twice with phenol:chloroform (1:1 by volume). The low molecular weight DNA was recovered by ethanol precipitation, resuspended in 25 μl Tris-EDTA, and treated with RNAse A for 2 h at 37°C prior to electrophoresis on 1.8% agarose gel.

### Western blots

Total cell lysates were prepared by lysing cells in lysis buffer (50 mM Tris pH 7.4, 5 mM EDTA, 250 mM NaCl, 50 mM NaF, 1 μg/ml leupeptin and aprotinin) at 4°C for 20 min. The protein concentration of supernatant was determined by Bio-Rad protein assay. Equal amount of protein was resolved on 8–10% SDS-PAGE gels and transferred to Immobilon P (Millipore). Membranes were blocked with 5% non-fat milk and probed with antibodies against p53, Rb, p21, myc, cyclin A, cyclin E, cdc2, bcl-2 (Oncogene Science), cdk2 (Upstate Biotechnology). After incubation of blot with horse-radish peroxidase-conjugated secondary antibody, reactive polypetides were detected by the enhanced chemiluminescence (ECL) system (Amersham).

### Hl kinase activity

Cyclin A, cyclin E, cdc2 and cdk2 associated kinase activities were measured *in vitro* after immunoprecipitation, using histone (Boehringer Mannheim) as a substrate. Total cell lysates were made from 300 μM PALA treated cells at indicated time points. For each immunoprecipitation 50 μg of protein saturated with proper antibody was incubated with protein G+A agarose beads (Oncogene Science) overnight at 4°C. After extensive washing in lysis and kinase buffer the beads were assayed for 30 min at 37°C in kinase buffer (50 mM Tris pH 7.4, 10 mM MgCl_2_, 1 mM DTT, 50 μM ATP, 1 μCi of (γ^32^P-ATP) containing l mg/ml substrate. Kinase reactions were analyzed by autoradiography following 12% SDS-PAGE and quantitation with the help of phosphorimager.

### E2F1 DNA binding activity assay

Cells were washed in PBS containing 1 mM PMSF, and then harvested to prepare nuclear proteins for assessing E2Fl DNA binding activity by gel retardation assay (46). Harvested cells were washed in wash buffer (20 mM HEPES, 2 mM DTT, 2 mM MgCl_2_, 1 mM PMSF, 2 μg/ml leupeptin, 1 μg/ml aprotinin) and lysed in wash buffer containing 0.2% Triton X-100 by incubation on ice for 10 min. Nuclei were then collected by centrifugation at 13,000 rpm for 10 sec at 4°C. The nuclei were resuspended in 50 μl of nuclear extraction buffer (20 mM HEPES, 2 mM MgCl_2_, 2 mM DTT, 420 mM KCl, 25% glycerol, 1 mM EDTA and protease inhibitors) and incubated on ice for 20 min. Cellular debris was removed by centrifugation at 13,000 rpm for 5 min at 4°C. The protein/DNA interaction assay was performed by preincu-bating 5 μg of nuclear proteins in a binding buffer (4% Ficoll 400, 20 mM HEPES pH 7–9, 2 mM MgCl_2_, 0.5 μg of salmon sperm DNA and 100 mM KCl) for 10 min at room temperature. γ^32^P-ATP labeled E2 oligonucleotide probe (10 pmol) was then added and incubated at room temperature for 30 min. The reaction products were resolved on a 4% polyacrylamide gel in 0.5 × TBE run at 160 V at 4°C for 2–3 h. Gels were dried and exposed to X-ray film.

### Brl transfection with pMTCyc A

Cells bearing a Zn^2+^ responsive cyclin A construct were generated by transfecting the pMTCyc A plasmid into Brl-3pr1 cells (Br-l pMTCyc A). pMTCyc A contains a 2.3 kb *Eco*R1 fragment of human cyclin A cDNA downstream of a sheep metallothionein promoter and a neomycin-resistant marker gene. Thirty randomly selected clones were expanded and screened by southern blot analysis of total genomic DNA with cyclin A probe. Four positive clones were analyzed further to see inducibility of the cyclin A transgene. Two of these clones showed distinct-induction of cyclin A expression in the presence of 100 μM Zn^2+^. Expression of induced cyclin A in the clones was determined after induction with 100 μM of Zn^2+^ for different time intervals. Total cell lysates were prepared and Western blot analysis was done as described above. Oligonucleosomal DNA fragmentation analysis of clones was done as described above after treatment with 300 μM PALA for 48 h and simultaneous induction of cyclin A with 100 μM Zn^2+^ for 9 h.

## Results

### Growth rate analysis in L-2 and Brl-3prl

**Figure 1:**
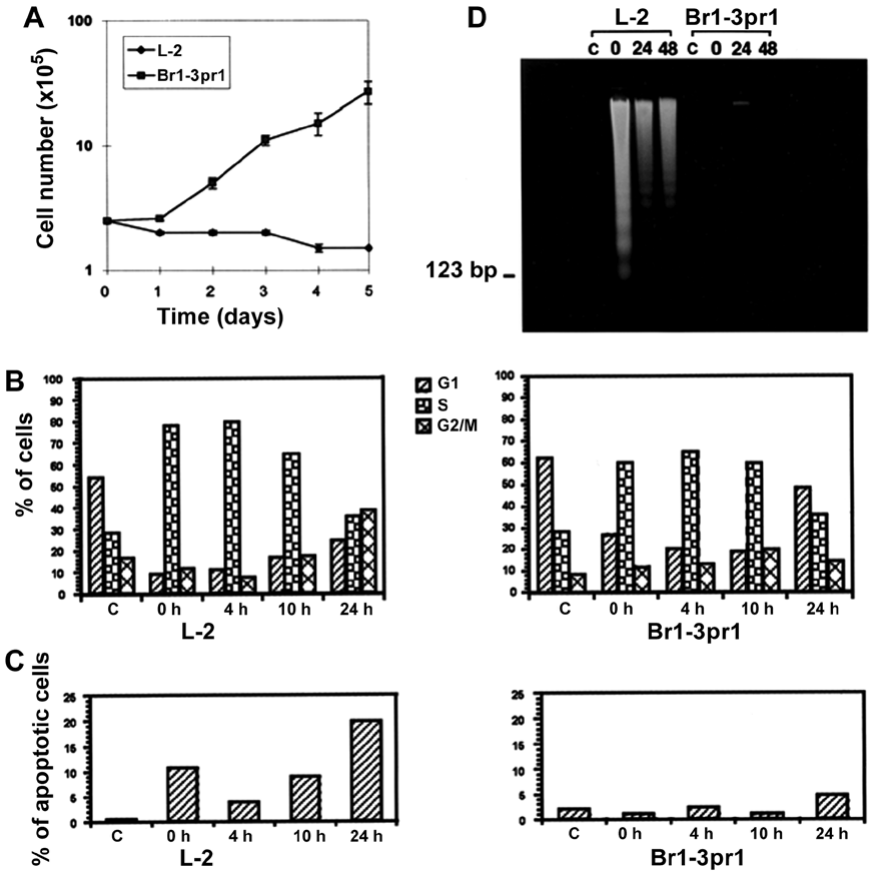
Cytotoxicity analysis of 300 μM PALA treatment on L-2 and Brl-3pr1 cells. (A) Growth inhibition of PALA treated cells analyzed by trypan blue dye. (B) Cell cycle analysis in the two variants prior to treatment (C) or after 48 h treatment with PALA at the indicated times. (C) Percentage of apoptotic cells at different time points. (D) DNA fragmentation analysis of untreated (C) or PALA treated cells at different time intervals.

Effect of 48 h PALA treatment at 20xLD_50_ on the growth rate of the two cell types, was assessed over a 5 day period. L-2 and Brl-3prl showed clear difference in their growth potential following drug treatment (Fig. 1A). After an initial growth arrest in both cell lines, Brl-3prl recovered and continued proliferation whereas L-2 cells failed to grow. These results demonstrated that the differential levels of drug sensitivity in the two cell types were manifested in their markedly different proliferation pattern within the first 24 h after exposure to the drug.

### Cell cycle distribution pattern following PALA administration

We investigated how PALA exposure affected proliferative pattern through the cell cycle in the two cell types. Untreated cells of both variants showed a similar cell cycle distribution pattern (C in Fig. 1B). It was noted that both cell lines treated for 48 h showed progressive accumulation of cells in S phase. However, the difference in cell cycle stage specific distribution pattern of the cells became evident following release from PALA treatment of L-2 and Brl-3prl cells. At the end of 48 h treatment (or same as 0 h, Fig. 1B), cells were predominantly in S phase in both the cell lines, although slightly higher proportion of cells in S phase were noted in L-2 than Brl-3prl cells. At 4 h post-treatment S phase cell population showed further increase in the two cell types but at 10 h, there was a decline in the percentage of cells in S phase in both cases with increase in G2/M phase cells. At 24 h post-treatment cell cycle distribution profile of L-2 cells showed more cells in G2/M phase than in G1 or S phases. Brl-3prl cells, however, revealed a pattern similar to the one seen in untreated controls. In view of our findings that there was significant loss in proliferative capacity as well as decrease in the number of surviving L-2 cells after PALA treatment, we compared the percentage of apoptotic cells using the sub-G1 FACS assay in both cell types.

There was significant cell death detected in L-2 cells immediately after 48 h (11%, 0 h, Fig. 1C) PALA treatment. Following a lower incidence (4%) at 4 h, there was a second wave of apoptosis induction detected in the L-2 cells at 10 h (9%) and 24 h (21%). Brl-3prl cells on the other hand did not undergo significant apoptosis up to 10 h post-treatment but showed slight increase (5%) at 24 h post-treatment. These results suggested that both cell types underwent an initial S phase delay/arrest following PALA treatment. Except for a small percentage, most Brl-3pr1 cells recovered and proliferated but increasing number of L-2 cells progressively underwent apoptosis.

### DNA fragmentation assay

Apoptosis in response to PALA treatment was assayed by the presence of oligonucleosomal DNA fragments in the two cell types. No significant DNA fragmentation was observed in L-2 cells treated with PALA for less than 48 h. At the end of 48 h (time 0 h, Fig. 1D) significant DNA fragmentation indicative of apoptosis was observed in agreement with the flow cytometry data reported earlier. Brl-3pr1 cells did not reveal any significant DNA fragmentation following PALA treatment at all the time points checked. These results reveal that L-2 cells treated for 48 h with PALA show progressive accumulation of cells in S phase and activation of an apoptotic pathway leading to cell death. Brl-3pr1 cells on the other hand showed a transient S phase arrest and recovered to proliferate without significant evidence of apoptosis in these cells. L-2 cells from several dishes had to be pooled for all subsequent protein expression and kinase activity studies since massive apoptosis was induced in these cells following drug treatment.

### Effect of PALA on cellular nucleotides

To determine if the apoptotic response of these cell lines to PALA treatment was due to differential effect on the nucleotide pools, cellular NTPs were quantitated in control (untreated cells) and in cells incubated with PALA for 48 h. As presented in Table I, there was a significant reduction in the pyrimidine nucleotide pools in both cell lines after PALA treatment. CTP pool size was lowered to <8% in both cases (p=0.024 for L-2 and p=0.002 for Brl-3prl cells). There was >95% decrease in the concentration of UTP (p=0.018 and 0.0002 for L-2 and Brl-3prl cells, respectively). In contrast to pyrimidine nucleotides, there was 30–60% increase in the purine nucleotide concentration. These data clearly demonstrated that the action of PALA in both these cell lines was comparable resulting in similar reduction in the pyrimidine NTPs.

### Expression of cell cycle regulatory protein

**Figure 2:**
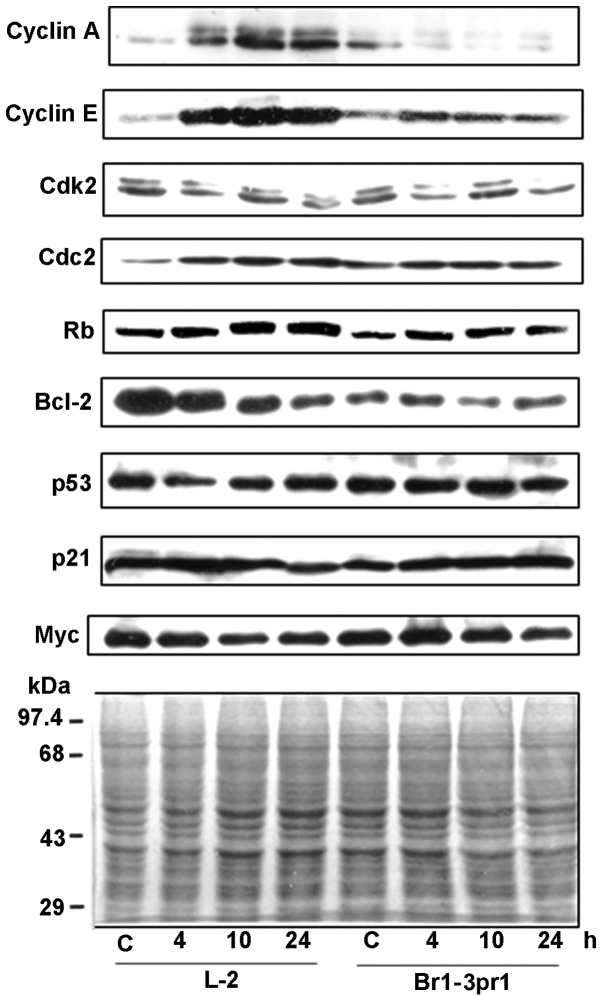
Expression of cell cycle regulatory proteins in cells after 300 μM PALA treatment. Total cell extracts were from untreated (C) and cells treated for 48 h with PALA and then released in drug-free medium for indicated times (4, 10 and 24 h). The Coomassie blue stained gel reflects the comparable amount of protein loading.

The expression profile of cell cycle and apoptosis regulatory proteins after PALA treatment were assessed to evaluate if they correlated with induction of apoptosis process. In order to assess the pattern of expression of cell cycle regulatory proteins in L-2 cells undergoing apoptosis and Brl-3prl cells recovering to proliferate following S phase arrest, western blot analysis of p53, p21, Rb, c-myc, cyclin A, cyclin E, cdk2, cdc2 and bcl-2 was performed at different time intervals after release from 48 h treatment of PALA (Fig. 2). Compared to the untreated cells (C), no apparent effect in the levels of p53, p21 and myc proteins were observed in the two cell types. There was moderate difference in the level of phosphorylated Rb proteins seen in the two cell types. L-2 cells showed increased accumulation of phosphorylated Rb compared with Brl-3pr1 cells. Marked increase in the amount of cyclin A protein was detected in the L-2 cells undergoing apoptosis with the highest level detected at 10 h post-drug treatment. In contrast, there was no increase in the level of cyclin A seen in the Brl-3prl cells. Cyclin E protein was found elevated in the L-2 cells and Brl-3prl cells compared to their respective controls (lanes C). The increase in cyclin E protein in L-2 cells despite being more than that seen in Brl-3prl cells was not as significant as that seen for cyclin A protein, at similar time intervals.

Expression of both cdk2 and cdc2 proteins were also monitored. Cdc2 protein amount was elevated in drug-treated L-2 cells compared to the control at all the time points checked. There was no distinct alteration in the level of cdk2 protein detected in the cell types. L-2 demonstrated a gradual decrease in the expression of bcl-2 from 4 through 24 h post-treatment; whereas bcl-2 expression levels remained virtually unchanged in Brl-3pr1 cells. Taken together these data document that in contrast to proliferating Br1-3pr1 cells, apoptosis in PALA treated L-2 cells is accompanied with discrete changes in the levels of some proteins. These include increase in the cyclin A and cdc2 along with decline in the bcl-2 proteins.

### Kinase activity assay

In order to determine if the levels of cyclins and kinases correlated with their associated kinase activities, immunoprecipitates of cyclin A, cyclin E, cdc2 and cdk2 were subjected to HI kinase assays. Quantitation of these data indicated a significant elevation in cyclin A, cyclin E associated kinase activities in L-2 cells at 4 and 10 h after PALA removal as compared to the control and there was relatively moderate increase in these kinase activities of Br1-3pr1 cells (Table II). Cyclin A and E are known to bind both cdK2 and cdc2, the catalytic subunits (20,21). Cdk2 and cdc2 kinase activities were markedly elevated in L-2 cells while in Brl-3pr1 cells, only cdk2 kinase was 3× higher post-PALA treatment. In L-2, cdk2 kinase activity was elevated ~9-fold at 4 h and 7.5-fold at 10 h while cdc2 kinase activity was ~1.6- and 2.1-fold elevated at these time points. The results indicate that higher cyclin A, cdc2 associated kinase activities correlated with higher expression levels of these proteins. Cdk2 kinase activity on the other hand was significantly elevated with no apparent increase in the amount of the protein in these cells.

### E2F1 DNA binding assay

**Figure 3:**
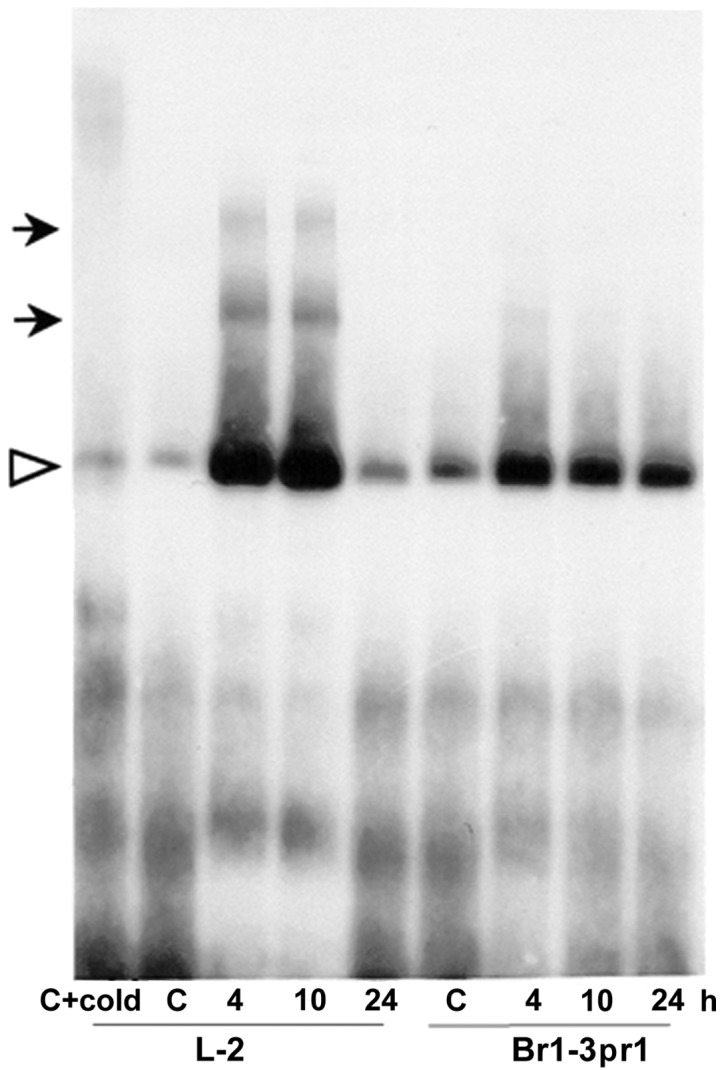
E2Fl-DNA binding. Nuclear extracts from untreated control (C) and 300 μM PALA treated cells, harvested at 4, 10 and 24 h post-treatment incubated with ^32^P labeled E2 oligonucleotide displaying E2F1-DNA complex (→).

Cyclin A kinase regulated E2F1 transactivation function has been proposed to represent an S-phase checkpoint that is activated in cells exposed to cytotoxic effects of drugs and ionizing radiation. We assayed for E2F expression and DNA binding activity in untreated and PALA treated cells by gel retardation assay. There was no difference in the level of expression of E2F protein detected in the treated and untreated cells (data not shown). As shown in Fig. 3, E2F complexes (bands shown with solid head arrows) were detected in L-2 cell extracts at 4 and 10 h post-treatment but were not seen in the control untreated or in the PALA treated Brl-3prl cells. It is important to mention that these L-2 cells also revealed progressively increasing incidence of apoptosis with enhanced cyclin A expression and associated kinase activities. At 24 h post-treatment sample, however, the gel retardation assay did not show discrete E2F complexes and only the nonspecific band (shown with open arrowhead) similar to untreated control cell extracts was seen. This might be explained by the fact that at this time, extensive apoptosis in treated cells may have eliminated most of the cells containing E2F with binding affinity for the cognate promoter sequence.

### Induction of apoptosis and E2F1-DNA binding in pMTCyc A transfected Brl-3prl cells after PALA and Zn^2+^ treatment

**Figure 4:**
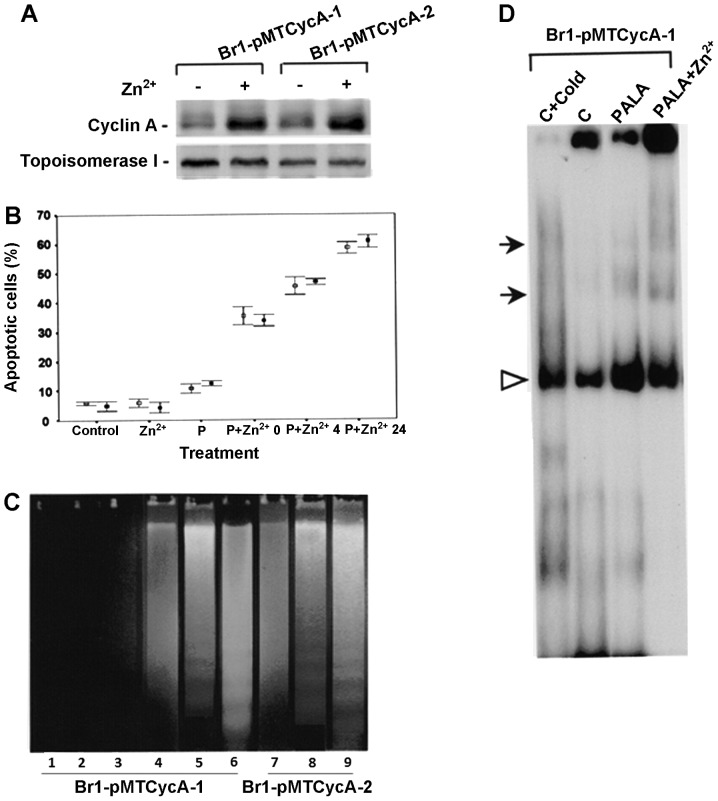
Characterization of inducible cyclin A expressing cells. (A) Cyclin A expression analysis. Brl-pMT Cyc A-1 and Br1-pMT Cyc A-2 cells were treated with 300 μM PALA for 48 h with simultaneous induction of cyclin A with 100 μM Zn^2+^ for 9 h. (B) Percentage of Br1-pMT CycA-l (open squares) and Br1-pMT CycA-2 (solid squares) cells undergoing apoptosis in controls, Zn^2+^ alone, PALA alone and PALA + Zn^2+^ at different time intervals. (C) DNA fragmentation in Br-l pMTCyc A-I and Brl-pMTCyc A-2 cells: Lane 1, Control; 2, Zn^2+^ treated; 3, PALA treated; 4–9, PALA + Zn^2+^ treated and harvested at 0 h, lanes 4 and 7; 4 h, lanes 5 and 8; and 24 h, lanes 6 and 9. (D) E2F1 DNA binding assay in BR 1-pMTCyc A-1 cells: Nuclear extracts from control, 300 μM PALA and 300 μM PALA + Zn^2+^ treated cells.

In order to confirm whether elevated expression of cyclin A in DNA antimetabolite treated cells can mediate or accelerate apoptotic response, stably transfected and conditionally over-expressing cyclin A cell lines were generated by tranfecting Brl-3prl cells with pMTCyc A (Br-lpMTCyc A). Cyclin A expression was induced in Br-lpMTCyc A cells by 100 μM Zn^2+^. Highest level of cyclin A protein expression was consistently achieved after 9 h induction with 100 μM Zn^2+^ in the clones tested. Fig. 4A demonstrates the inducibility of cyclin A protein in two different clones of Br-lpMTCyc A cells by 100 μM Zn^2+^ for 9 h. Cell viability was checked after treatment of cells with 300 μM PALA for 48 h and simultaneous induction of cyclin A with 100 μM Zn^2+^ for 9 h in the two clones (Br-lpMTCyc A1, Br-lpMTCyc A-2). Cell death was seen at all the time points, with the percentage of apoptotic cells being maximum at 24 h post-treatment (Fig. 4B). Apoptotic death was further confirmed by DNA fragmentation analysis (Fig. 4C). A time-dependent increase in the DNA fragmentation was noted in both clones from 0–24 h; with maximum fragmentation seen at 24 h. In contrast neither controls nor Zn^2+^ treated cells displayed presence of oligonucleosomal DNA fragments. Transfected cells, after 48 h PALA treatment, undergoing apoptosis were analyzed for E2F1-DNA binding activity also by gel retardation assay. As shown in Fig. 4D, E2F-DNA complex was clearly detected in cells induced to express cyclin A in parallel with PALA treatment. Low level E2F binding was also seen in cells treated with PALA in the absence of Zn^2+^. The data suggested that, even in the absence of Zn^2+^ induction, slightly elevated cyclin A expression due to leaky regulation of the promoter in the transfected expression vector may have caused low level E2F DNA binding in these cells.

## Discussion

Resistance to chemotherapeutic drugs remains largely unresolved and a germane issue of cancer biology. Molecular events which influence cancer cell sensitivity to chemo therapeutic drugs may include components involved in apoptosis and DNA repair pathways. It has been hypothesized that loss of apoptotic response often leads to chemotherapy resistance in cancer cells. Distinct induction of apoptosis in the L-2 cells and the absence of similar response in the Br1 cells demonstrated that the varying sensitivity to antimetabolite PALA observed in these two cell types was due to the difference in the drug-induced apoptotic response elicited.

It has been established that PALA cytotoxicity is mediated through its inhibitory action on the pyrimidine biosynthetic pathway and TAp73-dependent expression of Noxa and Bim (22–24). Hence differential action of PALA on the pyrimidine nucleotides in these cell lines could result in the observed contrast in apoptotic response. To determine if the action of PALA varied in these cell lines, intracellular ribonucleotides were analyzed after incubating cells for 48 h with (20xLD_50_) 300 μM concentration of the drug. This treatment resulted in a >90% decrease in pyrimidine pools, yet the extent of decline was similar in both cell lines. These data and the fact that the isogenic cell lines (Brl cells and cyclin A transfected Brl cells) also varied in their apoptotic response to PALA suggest that this difference is not due to the PALA-mediated alteration in the pyrimidine pools.

Expression of genes such as p53, bcl-2, c-myc and cyclin A are considered critical determinants of apoptotic response in cells (24–27). Cyclin A associated kinase activity has also been reported to be essential for paclitaxel sensitivity of human breast and ovarian cancer cells (28). It has been reported earlier that loss of p53 function in cells leads to resistance to cytotoxic treatments (29,30). Presence of an identical homozygous mutation in p53 in the two cell types (unpublished data) and lack of any perceptible alterations in the expression pattern of the mutant protein following drug treatment suggested that the apoptotic pathway, preferentially activated in the sensitive L-2 cells, was p53 independent.

A number of antitumor agents preferentially induce apoptosis at specific phases of the cell cycle (31–33). Cells undergoing apoptosis frequently do so in late G1 or early S in response to a wide range of stimuli (34–36) indicating that the gene products expressed at these stages may be involved in the activation of apoptotic pathway. Many of the gene products which appear to control apoptotic pathway are regulators of cell cycle progression; thus, cell cycle control and cell death appear to be tightly linked processes (37). Inappropriate activation of p34cdc2 kinase has been reported to lead to apoptosis (38). More recently dominant negative mutants of cdc2, cdk2 and cdk3 were found to suppress apoptosis, induced by diverse agents (39), known to be mediated by the active cyclin A kinase complex (40). Elevation in cdk2 and cdc2 kinase activities in L-2 cells undergoing apoptosis therefore reflected their involvement in activation of the apoptotic pathway. Activation of these kinases following induced cyclin A expression in Br-1pMTcyc A cells also suggested that failure to activate cyclin A kinase checkpoint in PALA treated cells allowed Br-l cells to escape apoptosis and proliferate. Induction of apoptosis has earlier been associated with activation of cyclin A dependent kinases but not activity associated with cyclin E or B (40).

It has been reported that overexpression of cyclin A alone in BHK cells cause ‘mitotic catastrophes’ with chromosomal fragmentation that resembles apoptosis (41). Cyclin A protein has also been implicated in myc-induced apoptosis (27). While our study provides strong evidence in support of cyclin A being a critical determinant of drug-induced apoptosis response, lack of correlation between cyclin A and myc expression suggests that cyclin A mediated apoptotic pathway may be myc independent in these tumor cells. It is however noteworthy that cyclin A kinase activation correlated with higher activity of cyclin E kinase in the L-2 cells. This result may be explained in view of the earlier observation that cyclin E kinase activity precedes and mediates upregulation of cyclin A kinase (42).

Enhanced cyclin A kinase induced apoptotic response, described in this paper, presents an apparent paradox to the concept that cyclin A underlies an S phase checkpoint that acts through phosphorylation of E2F1, to eliminate E2FI-DNA binding. Phosphorylation of E2Fl regulated by cyclin A/cdk2 in S phase is known to reduce the ability of the E2F1-DPl heterodimer to bind to DNA and mediate transcriptional transactivation. Mutant E2FI lacking cyclin A/cdk2 binding domain and not phosphorylated by cyclin A/cdk2, prolongs S phase causing apoptosis and increased sensitivity to the cytotoxic effects of S phase specific agents (43). Inhibition of cyclin A/cdk2 activity causing reduced E2F1 phosphorylation was also shown to increase sensitivity of human tumor cells lacking pRb to various classes of anticancer drugs (18).

Induction of apoptotic response in the antimetabolite treated cells displaying high cyclin A in parallel with augmented E2F1-DNA binding detected in this study provide contradiction to the above findings and suggests that cyclin A kinase effect on E2F1-DNA binding involves a complex regulatory process. Since cyclin A/cdk2 activation as well as E2Fl transactivation are both required during Gl to S transition, it is likely that additional factor(s) such as those required for initiation and/or elongation of DNA-replication modulate the effect of cyclin A kinase on E2Fl and vice versa. It is relevant here to mention that *in vitro* studies have convincingly demonstrated that addition of cyclin A alone reconstitutes both kinase activity as well as DNA replication in mammalian S phase cell extracts and does so by acting at a stage prior to elongation of nascent DNA (44). Although targets of cyclin-dependent kinases at the replication origin remain to be established, the activation pathway may either involve phosphorylation of replication proteins or phosphorylation of downstream kinases. Enhanced expression of cyclin A in drug treated L-2 and Brl-pMTcyc A cells may therefore have activated such downstream effectors of DNA replication to stimulate the elongation process leading to an abortive S phase.

Our results further suggest that such enhanced cyclin A driven replication may also induce increased E2Fl-DNA binding in the cells leading to apoptosis. Low cyclin A activity in drug treated cells, on the contrary, may slow down the replication machinery due to reduced activity of the downstream effectors allowing the cells to complete DNA synthesis without activating an apoptotic response. It is conceivable that inappropriate activation of both E2F1 as well as cyclin A in drug treated cells may induce apoptotic response. Cyclin A kinase, however, may enhance or reduce E2F1-DNA binding based upon the status of effector proteins involved in DNA synthesis. We do not know if loss of apoptotic response seen in drug treated Br1 cells occurs due to downregulation of cyclin A gene expression at the transcriptional or translational level. Further elucidation of this mechanism for cyclin A and other cell cycle regulatory proteins involved in activation of drug-induced apoptotic pathways will help identify useful therapeutic targets to overcome drug resistance in tumor cells.

## Figures and Tables

**Table I:** Effect of PALA on ribonucleotides in L-2 and Br1-3pr1 cells.

	% of Control NTP, Mean ± SD	
	
NTP	L-2	Br1-3pr1
CTP	7.1±2.7	7.5±2.2
UTP	3.3±0.2	4.7±1.2
ATP	141.7±18.8	161.6±28.5
GTP	123.0±8.0	159.2±31.5

The changes in the levels of nucleotides in PALA treated cells are expressed as % of control values. Data points are mean and SD of three separate experiments.

**Table II:** Kinase activities in L-2 and Br1-3prl cells.

	L-2				Br1-3pr1			
		
Kinase	Control	4 h	10 h	24 h	Control	4 h	10 h	24 h
Cyclin A	1	3.1	2.2	1.2	1	1.4	2.0	1.8
Cyclin E	1	3.4	2.2	1.8	1	1.5	1.5	1.7
Cdk2	1	9.0	7.5	5.0	1	3.0	2.0	1.7
Cdc2	1	1.6	2.1	1.5	1	1.0	1.1	1.2

Densitometric quantitation of the relative changes in the kinase activities. Control values of both L-2 and Brl-3prl were taken as 1.0 for normalization.

## Abbreviations

PALA: N(phosphonoacetyl)-L-aspartate
Mtx: methotrexate
BHK: baby hamster kidney
Rb: retinoblastoma protein
PAGE: polyacrylamide gel electrophoresis
